# Uncovering Pharmacological Opportunities for Cancer Stem Cells—A Systems Biology View

**DOI:** 10.3389/fcell.2022.752326

**Published:** 2022-03-11

**Authors:** Cristina Correia, Taylor M Weiskittel, Choong Yong Ung, Jose C Villasboas Bisneto, Daniel D Billadeau, Scott H Kaufmann, Hu Li

**Affiliations:** ^1^ Department of Molecular Pharmacology and Experimental Therapeutics, Mayo Clinic, Rochester, MN, United States; ^2^ Division of Hematology, Department of Medicine, Mayo Clinic, Rochester, MN, United States; ^3^ Department of Immunology, Mayo Clinic, Rochester, MN, United States; ^4^ Division of Oncology Research, Mayo Clinic, Rochester, MN, United States

**Keywords:** cancer stem cells, cellular niche, tumor microenvironment, systems biology, immunotherapy, drug resistance

## Abstract

Cancer stem cells (CSCs) represent a small fraction of the total cancer cell population, yet they are thought to drive disease propagation, therapy resistance and relapse. Like healthy stem cells, CSCs possess the ability to self-renew and differentiate. These stemness phenotypes of CSCs rely on multiple molecular cues, including signaling pathways (for example, WNT, Notch and Hedgehog), cell surface molecules that interact with cellular niche components, and microenvironmental interactions with immune cells. Despite the importance of understanding CSC biology, our knowledge of how neighboring immune and tumor cell populations collectively shape CSC stemness is incomplete. Here, we provide a systems biology perspective on the crucial roles of cellular population identification and dissection of cell regulatory states. By reviewing state-of-the-art single-cell technologies, we show how innovative systems-based analysis enables a deeper understanding of the stemness of the tumor niche and the influence of intratumoral cancer cell and immune cell compositions. We also summarize strategies for refining CSC systems biology, and the potential role of this approach in the development of improved anticancer treatments. Because CSCs are amenable to cellular transitions, we envision how systems pharmacology can become a major engine for discovery of novel targets and drug candidates that can modulate state transitions for tumor cell reprogramming. Our aim is to provide deeper insights into cancer stemness from a systems perspective. We believe this approach has great potential to guide the development of more effective personalized cancer therapies that can prevent CSC-mediated relapse.

## Introduction

**FIGURE 1:**
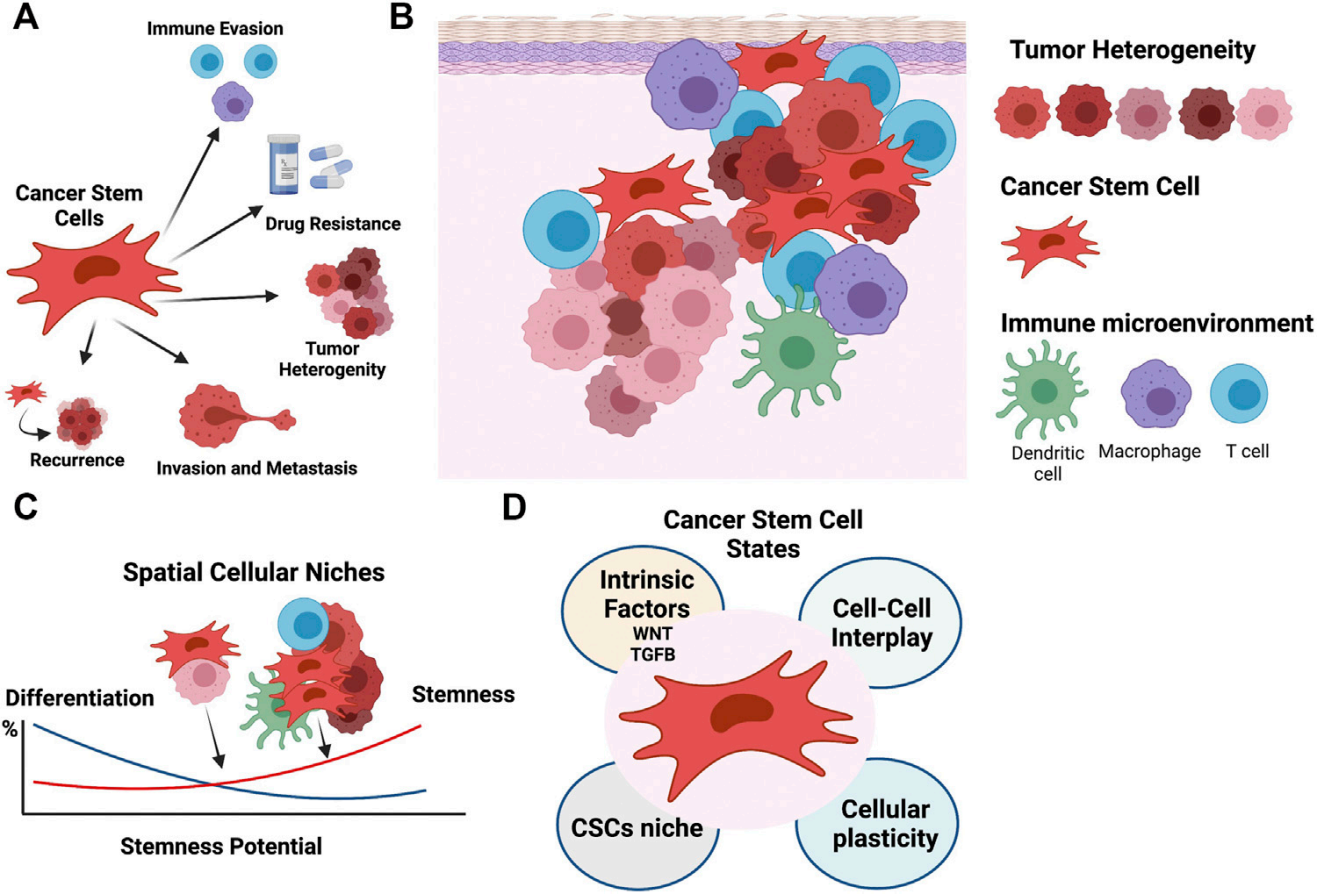
The cellular niche sustains the phenotypically diversity of cancer stem cells. **(A)** CSCs play fundamental roles in cancer, including contributing to tumor heterogeneity, and immune evasion, promoting migration and recurrence, and driving chemoresistance. **(B)** The CSC niche is supported by the tumor components, stroma, immune infiltrate, and tumor microenvironment. **(C)** Cell-cell interactions define spatial cancer niches and contribute to the phenotypically diversity of CSCs and cellular heterogeneity. **(D)** CSC phenotypic states are modulated by an array of factors, including intrinsic factors like WNT and TGFB pathway activation, cell-cell interactions, cellular plasticity, and the cellular niche. Figure created with BioRender.com.

In cancer, a gain of stemness can have profound implications on tumor aggressiveness, drug response and clinical outcome (Figure 1A). Here, we provide a systems biology overview of how the immune cell niche, cellular contexts, and molecular or genetic perturbations contribute to stem cell-like properties of malignant cells. We start by describing the sources of tumor heterogeneity and defining the cellular niche as a dynamic spatial domain harboring cancer stem cells (CSCs). We then elaborate on how this cellular niche is critical for communication between cell populations and move through the biological pathways endowing these stemness properties. We outline how systems biology approaches present an important strategy for identifying the crosstalk between immune cells, the bulk cancer, and CSCs. Finally, we discuss how these systems biology approaches open new avenues to dissect and manipulate cellular states and niches to impact cancer progression.

## Sources of Tumor Heterogeneity

Tumor complexity not only arises from the intrinsic diversity of the tumor cells themselves, but also from their interactions with other non-tumor cell types. The intratumoral heterogeneity of the cancer cells arises through mechanisms such as genomic instability, clonal evolution, cellular differentiation, and reversible plasticity. These contributions have been well studied, but most analyses fail to systematically characterize the contributions of other cells to cancer cell heterogeneity. Immune cells, which are also part of the tumor niche, present over 350 CD (cluster of differentiation) antigens, secrete close to 100 cytokines and chemokines, and belong to subtypes that express thousands of unique gene signatures (Davis et al., 2017). These immune cells, as well as other non-malignant stromal cells, co-exist with cancer cells, thus creating complex biological entities. Moreover, these stromal cells interact with and shape the evolution of the cancer cell populations (Figure 1B).

From a systems biology perspective, the contribution of each source of heterogeneity can be understood by dissecting cell populations, cellular niches, niche dynamics and the crosstalk between them. This will ultimately help devise disease spatial resolution maps that shed light on mechanisms of cancer progression and drug resistance. Such complex systems exhibit emergent properties that are not just the aggregation of unconnected individual behaviors, but are instead unique phenomena that reflect synergy in interactions between cells. The scientific discipline of systems biology aims to provide a quantitative and dynamic framework that leverages mathematical and computational techniques to unravel the complexity of cellular processes. This approach is applied with the view that a holistic view of systems will not only inform how a gene connects to a protein and an activity, but also help elucidate how a cellular phenotype is contingent on the biological context (where and when) (Kirschner, 2005). As such, systems biology approaches have the power to dissect the interplay between tumor and immune cells to characterize higher order properties and systems interconnectivity. For example, interrelationships between ligands and receptors have been an active area of research where a variety of system methodologies have been applied to define ligand/receptor co-expression, formation of multimeric complexes and cellular activity (see section entitled Probing the cellular niche: Technological advances). The large breadth of ‘omics data now available is enabling systems approaches to give new insights. Here, we will highlight the potential of systems biology to dissect the determinants of cancer stemness present in the cellular niche.

## Stemness of Cancer Cells

Stemness, defined as the potential for self-renewal and differentiation from the cell of origin, was originally attributed to normal embryonic stem cells that give rise to all cells in adult organisms. However, it has long been known that cancer cells show markers and properties of embryonic stem cells (Friedmann-Morvinski and Verma, 2014). Cancer progression often involves the gradual loss of a differentiated phenotype and acquisition of progenitor or stem cell-like features. Undifferentiated primary tumors are more likely to result in metastasis to distant organs, causing disease progression and poor prognosis, as well as resistance to available therapies. Within a tumor, phenotypic diversity and spatial cellular variation may also impact expression of key embryonic stem cell regulators, resulting in distinct paths to cancer stemness (Figure 1C).

## Systems Biology and Cancer Stem Cell States

### Dedifferentiation of Cancer Cells

**FIGURE 2:**
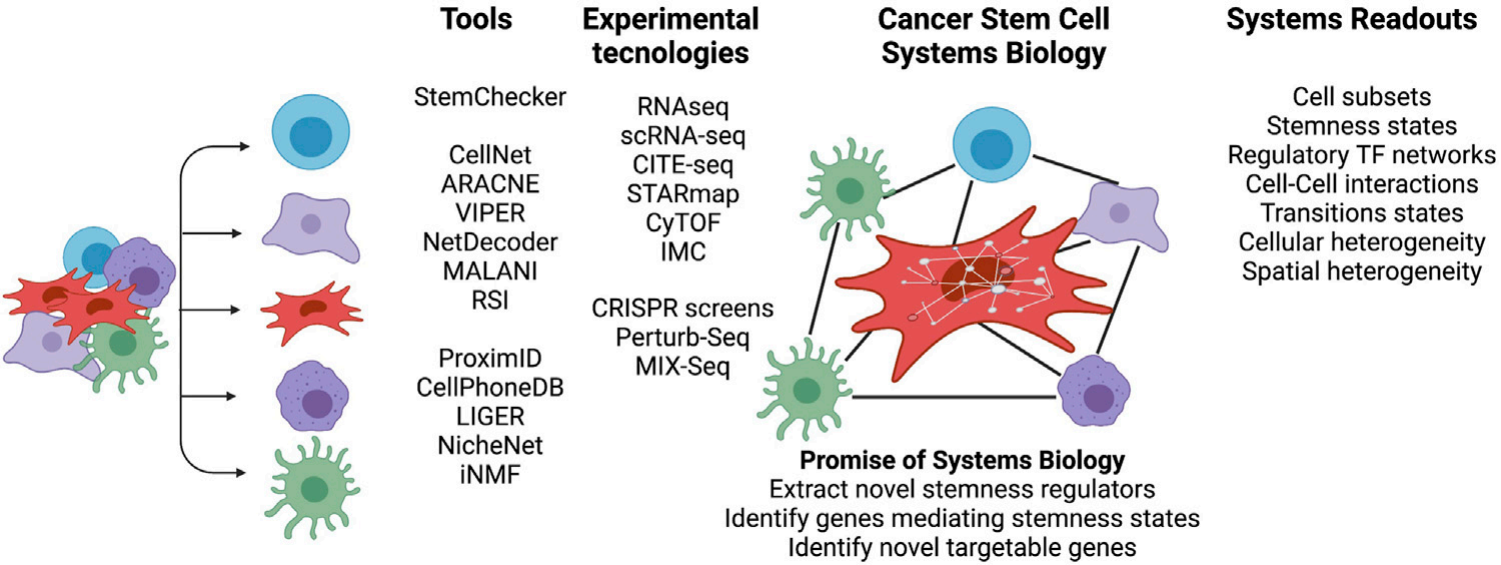
Cancer stem cell systems biology—Novel insights gained by integrated analyses. Overview of breadth of high-throughput technologies and tools to characterize the cross talk between CSCs, immune and tumor cells in a cellular niche. The niche cell subsets can be probed, for example, using bulk and single cell RNA-seq or cytometry by time-of-flight (CyTOF). CITE-Seq, STARmap and IMC (imaging cytometry) can spatially probe the cellular niche. A variety of systems tools provide a framework to interrogate signaling pathways, derive regulatory TF networks and understand the diversity of CSC phenotypes and their niches. Figure created with BioRender.com.

Although CSCs exhibit the stem cell-like properties of self-renewal and differentiation, they do not necessarily originate from the transformation of normal tissue adult stem cells (Friedmann-Morvinski and Verma, 2014). Oncogenic hits initiating malignant transformation may over time lead to a more dedifferentiated state and contribute to tumor cell heterogeneity. For instance, reverse engineered gene regulatory networks (GRNs) reconstructed by methods such as mode-of-action by network identification (MNI) approach, were used in our earlier studies and allowed the identification of HOXA1 as a key modulator capable of reversal phenotypes in breast cancer (Brock et al., 2014). Such inducibility of tumor plasticity suggests that CSCs do not necessarily originate from normal stem cells. Instead, under certain circumstances, cancer cells can dedifferentiate and acquire cancer stem cell (CSC)-like properties (Lyssiotis and Kimmelman, 2017). Here, the microenvironment of a tumor provides ample molecular cues and opportunities for cell-to-cell signaling to modulate the epigenome and phenotypic stem cell-like programs in cancer cells, frequently independent of their genetic backgrounds (Figures 1C,D) (Gingold et al., 2016). A variety of systems biology approaches, which are able to dissect transcriptional programs in various cellular states (Huggins et al., 2017; Luke et al., 2019), can also be used to understand CSCs (Figure 2). For instance, CellNet (Cahan et al., 2014) can reverse engineer GRNs to find transcription factors that are crucial in cell state maintenance. Others, like ARACNe (Basso et al., 2005) and VIPER (Alvarez et al., 2016), utilize GRN reverse engineering approaches to enrich for cellular regulons and identify regulatory genes that determine specific biological conditions. As an example, a set of systems biology tools developed in our lab have the potential to provide deeper look into CSC biology. For example, NetDecoder (da Rocha et al., 2016) builds a context specific network based on prior biological knowledge and enables genome-wide modeling of signal flows to extract genes that are critical under a specific biological context. Alternatively, Machine Learning Assisted Network Inference (MALANI) (Ghanat Bari et al., 2017), a machine learning-based method, creates *de novo* biological networks and has the ability to extract “dark genes” that are neither differentially expressed nor mutated but can play important roles in CSCs’ stemness. Moreover, Regulostat Inferelator (RSI) (Ung et al., 2019) searches for gene pairs that act like biological rheostats and can modulate phenotypic transitions in cancer cells, for example, the acquisition of stemness properties.

### WNT-β-Catenin Signaling and Stem Cell-like Phenotype in CSCs

Canonical WNT is a major pathway that regulates CSCs and induces stemness in several cancers (Vermeulen et al., 2010; Nguyen et al., 2019). The hallmark of this pathway is the activation of β-catenin-mediated transcriptional activity. WNT canonical pathway signaling is initiated by the binding of a WNT family protein to cell surface receptors to activate signal transduction (Kalbasi and Ribas, 2020; Yang et al., 2016). In the presence of WNT ligand, β-catenin evades proteasomal degradation, translocates to nuclei and activates transcription. This has several consequences for the CSC niche: 1) Increased phenotypic heterogeneity, 2) spatial diversity, and 3) impact on *de novo* immune response (Holtzhausen et al., 2015; Spranger et al., 2015; Luke et al., 2019) [for review see (Galluzzi et al., 2019)]. In particular, heterogenous activity of WNT has been observed in colon cancer, with high activity seen in regions close to stromal components (Vermeulen et al., 2010) (Figure 1D). Moreover, WNT-β-catenin pathway activation has been associated with immune exclusion of dendritic cells (DC) and T-cells from melanomas (Spranger et al., 2015; Spranger and Gajewski, 2015) and other cancers (Luke et al., 2019). With a reduction of CXCL9/10, CCL4 and other chemokines, recruitment of DCs and cross-priming of effector T cells in tumors is limited (Spranger et al., 2017). Further, DCs are re-wired to a regulatory state that is immune tolerant (Hall et al., 2011).

While WNT signaling in tumor cells is associated with a worse outcome, WNT signaling in the lymphoid compartment appears to modulate anti-tumor responses. In particular the WNT-β-catenin pathway regulates TCF1, a transcriptional factor that plays a critical role in T cell differentiation. TCF1 acts by biasing the differentiation of naïve T cell to CD4^+^ T helper subsets (Th1, Th2, and Th17). Moreover, WNT-β-catenin signaling promotes the generation of memory T cells, whereas the expansion of naïve CD8^+^ T cells and differentiation of effector T cells are inhibited (van de Wetering et al., 1991; Bienz and Clevers, 2003; Luke et al., 2019) [See review (Wang B et al., 2018) for in depth description]. This and additional observations support the pursuit of WNT inhibitors and combinatorial targeting of the WNT-β-catenin pathway to improve clinical outcomes of patients to overcome primary, adaptive, and acquired resistance to immunotherapy (Wang B et al., 2018; Zhang et al., 2020). However, as suggested by Wang B et al. (2018), it remains to be determined whether this strategy can be translated into the clinical practice to ultimately help devise better individualized immunotherapy treatments for cancer patients.

Systems biology approaches are also used to interrogate signaling and phenotypical programs that sustain cancer stemness (Figure 2). For instance, Pinto et al. (2015) collected 132 stemness signatures using publicly available gene expression datasets, RNAi screen results, and Transcription Factor (TF) binding site data to generate an interactive web-based server (StemChecker) that reports the overlap of input genes with stem cell signatures and the targets of transcription factors. Conversely, Malta *el al*. used a variety of normal stem cells with one-class regression machine learning to extract stem signatures, which they then used to infer a stemness score in many cancers (Malta et al., 2018). These studies showed that WNT and TGFB signaling pathways act in a different range in CSCs compared to non-neoplastic stem cells.

In poorly differentiated tumors, overexpression of key embryonic stem cell regulators (e.g., NANOG, OCT4, SOX2, c-MYC) (Malta et al., 2018) was observed to correlate with poorer outcomes. Further analysis also indicated that dedifferentiation features are associated with 1) mutations in genes that encode oncogenes and epigenetic modifiers, 2) perturbations in specific mRNA/miRNA transcriptional networks, and 3) deregulation of signaling pathways (Malta et al., 2018). Overall, these observations highlight the need to consider the CSC niche as distinct from normal stem cell niches. For this reason, future analysis must take into account the co-existence of diverse cell states and embrace dedifferentiation as a path to cancer stemness.

## Promise of Probing the Cellular Niche

### Tumor Microenvironment

The tumor microenvironment is the ecosystem that surrounds tumor cells inside the body. It includes a variety of cell types, including immune cells, stromal cells, adipocytes, fibroblasts, and vascular cells, as well as extracellular vesicles (EVs), extracellular matrix and molecules produced and released by all of these cell types. These TME components are not just bystanders in the tumorigenic process, but instead play a decisive role in tumor differentiation, epigenetics, dissemination, immune evasion, and drug resistance (Labani-Motlagh et al., 2020). In particular, the cross-talk between tumor cells and cells in the TME fuels and shapes tumor progression, giving rise to dynamic and complex ecosystems in both primary and metastatic sites.

Because most cancer deaths result from the development of distant metastasis, it is important to decode the dynamic interactions between cancer cells and the TME in individual sites during tumor development, progression, and response to therapy. For example, metastatic cells can differ from neoplastic cells at the primary site in key ways as they adapt to the unique metabolic conditions in the metastatic site (e.g., Ferraro et al., 2021), acquire mutations, evolve independently, and persist despite exposure to therapy. However, our ability to infer the cell-cell communications and investigate cellular plasticity in metastases is limited by the often simplistic models or is built on knowledge gained from the primary tumor. Therefore, developing models that focus on investigating the immune and transcriptional landscapes as well as cellular cross-talk by screening distant metastasis will help to better characterize dormant micro-metastases and identify new therapies to target metastatic tumors.

In current studies designed to understand the role of TME in the transition of ductal carcinoma *in situ* (DCIS) to invasive breast cancer (IBC) and progressive disease, Risom et al. used a multicompartmental analysis to compare functional biological states during tumor progression. Tumor invasiveness was correlated with a higher number of cancer associated fibroblasts (CAFs) and density of fibrillar collagen, a shift from monocytes to antigen presenting cells (APCs) and intraductal macrophages, and increased density of T and B cells in stromal compartments. These findings highlight a model in breast cancer (BC) where invasiveness occurs through the dynamic interactions with surrounding stroma and immune cells with the epithelial compartment of the tumor (Risom et al., 2022). Further, in a recent clinical trial (Hurvitz et al., 2020), BC patients were randomized to receive three distinct anti-HER2 treatments (trastuzumab, lapatinib, trastuzumab + lapatinib) followed by six cycles of standard combination chemotherapy along with the same anti-HER2 therapy. Serial analysis of the pre- and post- treatment demonstrated an increase of immune and stromal signatures after HER2 targeted-therapy alone but decreasing strengths of these signatures after addition of chemotherapy to the HER2 targeted-therapy. In particular, there was a reduction of M1 macrophages and increase in CD8^+^ T cells (Hurvitz et al., 2020). Collectively, these findings support the view that the TME changes over time, highlighting the possibility that factors converge to select the most adaptable tumorigenic cells and ecological environments for the tumor to thrive and reach its metastatic potential.

As a result of metabolic differences from site to site (Ferraro et al., 2021), as well as likely differences in cell types and cellular products, the TME is increasingly viewed as a highly heterogenous milieu that varies across tumor sites or so-called niches (spatial heterogeneity). This complexity needs to be considered at a systems level to design better therapeutic options for cancer patients. For example, the identification of the tumor dominant immune evasion mechanism within the TME can inform on the best patient therapeutic approach (Sanmamed and Chen, 2018). On the other hand, tumor stiffness can dictate a drug’s ability to reach the tumor (Olive et al., 2009). Clinically, a major challenge to understanding the TME is the limited ability to capture sequential tissue samples from cancer patients. However, recent advances in three-dimensional (3D) platforms like organ on a chip and microfluidic devices, as well as the development of humanized mouse models or explant 3D cultures model (patient- or mouse-derived tumor spheroids) (Sanmamed and Chen, 2014; Zitvogel et al., 2016; Jenkins et al., 2018; Vunjak-Novakovic et al., 2021), can provide an excellent opportunity to bridge this gap. Collectively, these tools have been developed with the view that a better understanding of the interplay of bi-directional communication between the tumor and TME, and CSCs will help identify improved cancer therapies.

### The Cellular Niche and Tumor Microenvironment

The term “niche” is commonly used to describe an anatomically distinct regions within a tissue (or tumor). This description, however, fails to capture the interactions with surrounding cell populations and microenvironmental cues. Instead, a niche might be more properly viewed as a spatiotemporal dynamic state that is modulated by external perturbations to induce a permissive tumorigenic environment. Because tumor cells, stroma and immune cells are crucial determinants of malignant growth (Visvader, 2011; Plaks et al., 2015), understanding how a cellular niche responds to each cell type in the TME is crucial for successful therapeutic targeting. In particular, cell-cell communication mediated by surface receptor-ligand interactions is an attractive process for pharmacological intervention. Accordingly, more needs to be known about this cell-cell communication. Moreover, immune cells are known to rewire in response to external stimuli arising from physical interactions with neighboring cells and secreted ligands. The resulting communication between tumor and immune cells impacts tissue homeostasis and disease progression. For example, in high-grade serous ovarian cancer (HGSOC), cancer cell progenitors have been found to migrate from the fallopian tube to more distal locations where the cellular niche allows for phenotypic shifting and propagation (Ng and Barker, 2015). In this way the cellular niche is intimately tied to cancer stemness and *vice versa* (Figure 1B–D).

### Highly Dynamic Cellular States and CSCs

During tumor evolution, it appears that tumor cell-extrinsic factors (the TME) as well as tumor cell-intrinsic factors (e.g., epigenomic changes) influence cellular states. Recently, Marjanovic et al. (2020) and LaFave et al. (2020) showed that a diverse and continuous range of states exist in a model of lung cancer progression. These co-existing states, which captured lineage infidelity and cellular plasticity, exhibited features of drastically different cell types, suggesting the ability of cancers to explore a broad phenotypic space. Although some of the cells in these lung cancers resemble stem cells and CSCs in their ability for robust growth and differentiation potential, their phenotypic programs are distinct (LaFave et al., 2020). Thus, a key step to better understanding CSCs is to thoroughly characterize their transcriptional states and ability to switch between CSC and non-CSC states.

## Probing the Cellular Niche: Technological Advances

A challenge with CSCs is that only a small number of stem cell markers have been identified. This limited set of markers reflects the small proportion of CSCs generally present in tumors and the poor conservation of CSC surface markers across cancers. Therefore, from a systems perspective, an *ab initio* approach is best for the identification of CSCs.

A diverse array of high-throughput technologies has been used to zoom in on CSCs and their niches within cancer patients. The oldest and most common modality is RNAseq. A limitation of traditional bulk RNAseq, however, is that it averages expression across thousands of cells within a sample. Because of this limitation, understanding and constructing regulatory networks for distinct types such as CSCs has been difficult with bulk RNAseq alone.

There is, however, precedent for studying distinct tumor components using bulk RNAseq. CIBERSORT (Newman et al., 2015) has been used extensively to computationally dissect immune cell populations from these bulk samples; and several methods exist for constructing tissue level networks. CIBERSORT uses a knowledge-based signature matrix that encapsulates major 22 functionally defined human hematopoietic subsets to deconvolute and infer cell composition from gene expression profile data, utilizing support vector machine (SVM) regression methods. This approach has played a key role in decoding immune cell population, particularly in bulk solid tumors where tissue dissociation protocols and cellular enrichment techniques limit the efficacy of single-cell methods and provide only a partial view of the wider cell heterogeneity. Extending this approach in a new direction, a groundbreaking study by Thorsson *et al.* used TCGA RNAseq data across 33 cancer types and more then 10,000 patients to identify signatures for six TME subtypes and demonstrate that these TME subtypes can be associated with prognosis as well as genetic and immune modulatory alterations (Thorsson et al., 2018). Reflecting the importance of the TME to tumor behavior, the TME subtypes identified by Thorsson *et al.* were reportedly able to predict disease outcomes and help guide novel treatments. More recently, Bagaev *et al.* derived 29 expression signatures to establish four TME subtypes (immune enriched fibrotic, immune enriched non-fibrotic, fibrotic and depleted) that correlate with immunotherapy efficacy in melanoma (Bagaev et al., 2021). As demonstrated by these studies, it is possible to use RNAseq to study a subpopulation of cells (e.g., immune cells or stromal cells) if the transcriptional profile is detectable and distinctive. However, given the rarity of CSCs, it has been difficult to study CSCs themselves using bulk RNAseq. Moreover, while publicly available multi-omics signatures from non-neoplastic stem cells were previously used to derive a stemness score in tumors (Malta et al., 2018) that can then be applied using deconvolutional methodologies to identify CSCs in bulk RNAseq, a key caveat is that CSCs do not necessarily share the same transcriptional programs as normal stem cells counterparts. Taking a different approach, Aran *et al.* probed the normal tissue adjacent to the tumor (NAT), which is morphologically similar but phenotypically different from the tumor and focused on stromal pathways to query the interaction between adjacent tissues (Aran et al., 2017). These authors uncovered NAT-specific characteristics, namely activation of pro-inflammatory immediate-early response genes concordant with endothelial cell stimulation. Furthermore, previous studies on breast NAT suggested that the microenvironment surrounding the tumor, not the epithelial cells, is essential for understanding disease recurrence and developing surgical strategies (Graham et al., 2011). These studies have shown that by zooming into the tumor niche and its adjacent boundaries we can acquire deeper understanding of cell-cell interplay and dissect the phenotypic states that sustain stemness.

With the advent of single-cell RNAseq (scRNA-seq), new avenues to investigate and quantify molecular features at single-cell resolution have emerged. Single-cell technologies have the ability to directly evaluate cell states, heterogeneity, and lineages (Saelens et al., 2019). However, more effort is required to provide understanding of cell-cell interactions as well as their co-evolution and adaptation to perturbations in the cellular milieu, which is needed for the construction of a comprehensive cellular interaction map (Elmentaite et al., 2019). Current efforts to build a human body atlas (Regev et al., 2017) will provide important information on the baseline stromal, tumor and immune tissue heterogeneity. Tissue specificity is particularly important in immune cells because they both circulate and remain tissue resident; profiling of both populations can enhance understanding of which cells are recruited to become part of the TME. Despite the promise of scRNA-seq, it also has a key limitation: Enrichment and dissociation strategies that are used to separate single cells for profiling and capture cells of interest disrupt tissue organization and result in loss of spatial information (Elmentaite et al., 2019).

Single-cell computational frameworks have thus far focused in dissecting the ligand-receptor interactome based on expression of ligands and receptors across cell populations. The various computational frameworks take different approaches (Zhou et al., 2017; Cohen et al., 2018; Kumar et al., 2018). CellPhoneDB (Efremova et al., 2020) uses scRNA-seq to decode intercellular communication networks by inferring multimeric ligand-receptor interactions between cell states based on expression of a receptor by one cell state and a ligand by another cell state. By assessing which structural complexes are ubiquitously expressed in a tissue and not varied between cellular states, ligand-receptor cell specificity is assured. Because multi-subunit heteromeric complexes often swap subunits to ensure ligand specificity, expression of all subunits of a functional complex is required for downstream signaling. Vento-Tormo first applied CellPhoneDB to the study of early human pregnancy and interaction between fetal and maternal cells with a focus on trophoblast-decidua interactions that underlie common diseases of pregnancy such as pre-eclampsia and still births (Vento-Tormo et al., 2018). The authors uncovered receptor-ligand complexes that can modulate trophoblast differentiation. Moreover, they identified an association between three major decidual natural killer (NK) cell populations, their cell markers, and their expression of cytokines, chemokines and receptors, with different reproductive outcomes, providing a cell atlas to understand normal and pathological pregnancies (Vento-Tormo et al., 2018). In the context of CSCs, such a strategy could potentially be applied to dissect receptor-ligand interactions that help sustain stemness if achievable scRNA-seq resolution allows for the reliable identification of CSCs.

Using RNA velocity measurements that predict the fate of cell populations (splicing and non-splicing ratio) and provide a measure of stemness, we can potentially identify CSCs in a non-biased manner. Such strategy is particularly attractive when we consider the potential heterogeneity of the CSC compartment. This strategy was employed by Gautam et al. (2021) to generate a multispecies cell roadmap for human and porcupine ocular compartments. The ability to use organoids and enrich for stem cell like progenitors offers new possibilities to further decode CSCs.

Boisset *et al.* have manually microdissected 727 mouse interacting cellular structures and used scRNA-seq to create an interacting cellular network (Boisset et al., 2018). Using permutations, they have created a null distribution of random interactions between cell populations that permits identification of enriched or depleted interactions as compared to the background model. More recently, the authors of NicheNet have assembled prior knowledge on ligand-receptors and their signaling pathways using public resources and used expression datasets to generate weighted networks with regulatory scores that infer activity (Browaeys et al., 2020). Overall, these studies provide immense insight into the cell-cell interactome, but these studies all make several key assumptions: 1) That transcripts encoding receptors are translated into proteins that are translocated to the membrane, 2) that ligands are successfully transported out of the cells, and 3) that cells are in proximity with interacting partners in tissue space (Elmentaite et al., 2019). These approaches are now starting to be applied to extremely rare populations such as CSCs and yield great promise to depict their secretome.

Mass cytometry (cytometry by time-of-flight, CyTOF) has revolutionized human immune cell profiling by allowing the simultaneous measurement of >40 surface markers on a single-cell basis (Bracci et al., 2020). CyTOF is particularly valuable when analyzing samples with a limited number of cells such as tumor biopsies, longitudinal studies or response to therapy, e.g., in clinical trials (Gadalla et al., 2019). The high degree of multiplexing in the CyTOF is possible because antibodies are coupled to rare metal isotopes that provide a unique mass tag to each marker to overcome spectral overlap commonly observed in multiparameter flow cytometry. Recently, Gallad *et al.* validated CyTOF panel data against flow cytometry and demonstrated equivalent ability to identify cell subsets or perform phenotyping, but with smaller cell numbers (Gadalla et al., 2019). CyTOF barcoding strategies enable the marking cells from separate samples, which allows for the simultaneous measurement of multiple samples, thus eliminating multiple sources of sample-to-sample variability (Behbehani et al., 2014). CyTOF has been applied to characterize CSCs specifically in leukemias and lymphomas; however, this requires *a priori* knowledge of the proteins at the cell surface and available antibodies amenable to conjugation. More recent studies have used CyTOF in combination with CITE-Seq (cellular indexing of transcriptomes and epitopes by sequencing), thus creating a multimodal approach with simultaneous quantification of single-cell transcriptomes and surface proteins. For example, Yao *et al.* have demonstrated that B cell, monocyte/macrophage, and plasmacytoid dendritic cell abundance across three methods (scRNA-seq, CyTOF and CITE-Seq) is consistent, but T cell measurements have greater variability. Additionally, this trimodal analysis indicates that there is a good correlation across scRNA-seq and protein expression for highly expressed cell type markers (Yao et al., 2020). Because these three single-cell approaches enable the simultaneous identification of cell types, cell states and characterization of cellular heterogeneity at transcriptomic and/or protein levels, understanding the concordance of the measurements among these three modalities is of great interest. Collectively, these observations suggest that the of use multimodal omic datasets can open new avenues to dissect CSC-immune cell interactions.

A major limitation of these single-cell techniques is the lack of spatial transcriptomic information that is key for understanding the CSC niche. 3D intact-tissue RNA sequencing methods exemplified by STARmap allow for mapping of more than 1,000 genes in sections of mouse brain to define cell types and establish a roadmap for cell organization principles (Wang X et al., 2018). Alternatively, imaging cytometry (IMC) is emerging as a transformative technique that allows for multiparametric analysis of >40 protein markers in frozen and FFPE (formalin-fixed, paraffin-embedded) tissue samples (Chang et al., 2017; Bouzekri et al., 2019). IMC provides a unique window into structural features of the tissue under investigation, validates spatial ligand-receptor interactions and identifies the distribution of cell types within the tissue to build a spatial map (Bodenmiller, 2016). In breast cancer, for example, clear differences have been noted in markers like cytokeratin, HER2, E-cadherin and c-MYC within single tumors, highlighting the intra- and interpatient heterogeneity that can be captured with IMC (Giesen et al., 2014; Wagner et al., 2019). As a result, IMC has the ability identify each cell type in its environmental cellular context, thereby providing a unique spatially resolved view of the tissue and allowing more accurate inference of cellular functional states. The downside is that this approach is not yet suitable for high-throughput screening of ligand-receptor interactions due the limited scanned tissue area (1 μm), high sample cost, and paucity of computational platforms available for data analyses. Bodenmiller *et al.* have developed multivariate computational tools to visualize and analyze multiplexed images of human tissue sections generated by IMC (Schapiro et al., 2017). Another platform recently proposed by Greenald *et al.* utilizes a library containing a large collection of manually curated cells, TissueNet (one million cells from six organs and different imaging platforms) and a deep learning model to achieve human-like cell and nuclear segmentation (Greenwald et al., 2021).

Future IMC studies focusing on a well-defined tissue sections that can be layered with ‘omic information will be of great interest for dissecting the interplay of between cell populations (Bodenmiller, 2016). Whether IMC layered with ‘omic data can also inform studies of rare cells such as CSCs remains to be seen.

## Future Directions in Manipulating the Cancer Stem Cell Niche

### Biological Perturbations to Modulate the Cellular Niche

A systems biology strategy to decode mechanisms underlying CSC cell fate decisions involves challenging stemness regulatory networks with external experimental perturbations. Upon acquisition of unimodal and bimodal single cell omics or matched multi-omics, cell fate control decisions can be inspected to define robust cell states and novel regulators that control stemness transitions. Experimentally, this can be achieved using RNAi knockdown and CRISPR-based knockout experiments. High throughput methods that generate large perturbation datasets like the Connectivity Map (CMAP) are limited in their biological contexts (Lamb et al., 2006; Bush et al., 2017; Ye et al., 2018). Conversely, Perturb-Seq combines pooled perturbation screens with scRNA-seq and cellular readouts to assay many cells within a mixed culture (Dixit et al., 2016). MIX-Seq, a more recent approach, provides the ability to pool hundreds of cancer cell lines and co-treat them with one or more perturbations, simultaneously profile the cells’ readout using scRNAseq, and resolve the identity of each cell based on single-nucleotide polymorphism (SNP) profiles (McFarland et al., 2020). Application of these exciting new approaches will undoubtedly expand current understanding of how genetic and phenotypic perturbations impact cells in different contexts, providing new tools that can potentially be applied to CSCs and their cellular niche.

## Systems ‘Omics Integration

With the sea of available ‘omic data, there is an urgent need for multi-omic data integration to provide systems-level information. Clustering of multi-omics has been implemented at various steps in the data analysis pipeline (e.g., early *vs*. late integration) [see reviews (Ramazzotti et al., 2018; Rappoport and Shamir, 2018)]. A drawback to all these strategies is that all data are treated equally, which may not reflect the biology of a disease (Ramazzotti et al., 2018). Recently, Stuart *el al*. suggested harmonizing single cell data across distinct modalities by selecting anchors (a common set of features) between datasets to recover matching cell states (Stuart et al., 2019; Stuart and Satija, 2019). A second key challenge is that some methods implicitly assume that data heterogeneity arises mostly from technical variation and is of no biological importance. To overcome this limitation, LIGER (linked inference of genomic experimental relationships) uses integrative non-negative matrix factorization (iNMF) for data reduction combined with joint clustering to define a cell label and assemble a neighborhood graph (Welch et al., 2019). Gao *et al.* have subsequently expanded LIGER capabilities to iteratively incorporate multiple data modalities and massive datasets (Gao et al., 2021). Overall, these strategies recover cell states but do not zoom in deep enough to capture the interplay between cells or levels of ‘omic data.

To dissect the cellular niches, future strategies will need to integrate ‘omic data in a way that achieves a better understanding of cellular states and their contributions to the diversity of CSC phenotypes. Such innovations will be key if we are to design novel therapeutic strategies that can disrupt key cell-cell communications and lead to the demise of CSC populations.

## Population Dynamics

Single cell experiments allow the study of tumors and their heterogenous cell populations that often coexist in an evolutionary continuum. Pseudotime trajectory methods (also known as trajectory inference methods) (Trapnell et al., 2014; Haghverdi et al., 2016; Setty et al., 2016; Saelens et al., 2019; Wolf et al., 2019) allow for the placement of cell populations within a trajectory based on their expression profiles. Because most single cell datasets are static and represent a unique snapshot in time, these cellular trajectories represent more a cellular states axis rather than real time, while encapsulating information from past and present. A challenge with the current pseudotime methodologies is that multiple dynamics can lead to the same cellular “state.” For example, cellular fate decisions such as cell death, proliferation, or differentiation can result from multiple causes. In order to understand the origin of these fates, we need to know the directionality of the biological process. Our ability to merge several biological snapshots in time, for example, from time series data, can help to decode the range of “real” existing states. Otherwise, the exploration of cellular states is currently being addressed in the field using dynamic inference methods. For example, Weinreb *et al.* used a dynamic inference framework named population balance analysis (Weinreb et al., 2018) that aims to limit the state search space by introducing biophysical constrains (cell density) to define cellular states. Conversely, Fischer et al. (2019) used RNA velocity information (spliced to unspliced ratio) as a mean to provide directionality to cellular trajectories. RNA velocity provides a measure of a cell’s internal compass (La Manno et al., 2018; Bergen et al., 2021) by quantifying nascent and mature messenger RNA. Here, we can also use matched multi-omic data integration (scRNA-seq and scATAC-seq) to identify key biological entities in each cell population and constrain the range of truly available cellular states to infer cell population dynamics. Future integration of multi-modal omics with spatial information derived from IMC will help to increase our spaciotemporal resolution, infer more accurate cell relatedness measures, and tackle key questions such as the extent of diversity of the CSC niche and which candidate targets are promising to reduce or re-wire CSC populations.

## Cancer Stem Cell Systems Biology—Emerging Insights Into CSC Stemness

Figure 2 summarizes representative systems biology approaches that can decipher stemness of CSCs. Current and new technological advances are essential to probe different layers of CSC regulation. Moreover, there is an urgent need for deep cell phenotyping using insights gained from integration of available systems approaches. Cell subsets can be investigated for *in silico* isolation of CSCs and used to explore the degrees of stemness defined by the combined actions of signaling pathway activities, cell-cell interactions, and TF regulatory networks to formulate phenotypic recipes that drive cellular states. To mirror the fact that CSCs are exposed to a myriad of cellular environmental stimuli, perturbation experiments open the possibly to study the impact of genetic changes, zoom into cellular rewiring, and identify cellular vulnerabilities. A combination of these approaches may overcome current limitations in targeting CSCs and allow the identification of novel targetable genes.

## Future Impact on Individualized Medicine

In summary, CSCs reside in tumor ecosystems composed of a plethora of cell types communicating in ways that drive cellular phenotypes. Therapeutics that target cell-cell interactions have become a useful tool in clinical practice and can be considered for CSC targeting (He and Xu, 2020). Key examples include ipilimumab, which targets the CD28/cytotoxic T lymphocyte antigen 4 (CTLA4) interaction, as well as pembrolizumab and nivolumab, which both target the programmed cell death 1 (PD1)/PD1 ligand 1 (PD-L1) interaction (Topalian et al., 2012). In addition to these FDA-approved immune checkpoint inhibitors, other inhibitory immunoreceptors that are being evaluated as potential targets for clinical intervention include LAG3, TIM3, TIGIT, B7H3, CD93, CD73, adenosine A2A receptor and BTLA (He and Xu, 2020). Despite the clear success of immune checkpoint inhibitors in several tumor types, response rates are limited (10–30%), especially in solid tumors (Hamanishi et al., 2015; Sarkar et al., 2016; Dempke et al., 2017; Disis et al., 2019; Palaia et al., 2020). The limited response rates seen with these agents are likely due to the complex network of interactions involving multiple cell types present in the TME, including CSCs, which leads to phenotypic heterogeneity we do not yet adequately understand (Sarkar et al., 2016). CSCs themselves exhibit immunosuppressive properties such as expression of inhibitory checkpoint ligands, low expression of MHC-I molecules and natural killer cell (NK cell) receptors, and low or absent expression of innate immune receptors, which renders the CSCs resistant to killing by a variety of immune cells (Bayik and Lathia, 2021). Inhibitory activity of immune checkpoints are determined by the cell surface levels of inhibitory receptors, ligand interactions with those receptors, turnover processes, and posttranslational modifications that regulate signal transduction (He and Xu, 2020). Therefore, most recently, CSC targeting has been carried out with combinations of dendritic cell-based vaccines, oncolytic viruses, and immune checkpoint blockades (for review see (Badrinath and Yoo, 2019)). Better understanding of potentially targetable ligand-receptor interactions involving CSCs has the potential to tailor these strategies. Furthermore, such understanding has the potential to expand the targetable interactions beyond tumor-immune cell interactions to include tumor-nonimmune stroma interactions as well.

Successful application of singe cell technologies and the integration of different modalities are paramount for better understanding of the factors that determine whether CSCs will respond to various therapies or not. Improved ability to quantify ligand-receptor interactions, dissect their spatial relationship (co-localization) and assess their association with outcome appear to be needed if we are to devise individualized CSC targeting. Indeed, current analyses suggest that for tumor-immune targeting there may not be a single predictor of clinical response. Instead, it is possible that the strengths of multiple ligand-receptor interactions will need to be assessed in addition to immune checkpoint components in order to predict response to immune checkpoint blockade (Ribas and Wolchok, 2018). Systems biology techniques are capable of extracting this biological information from ‘omic data and helping derive a systems view.

In contrast to CSC cell surface markers that are expressed on stem cells, intracellular stem cell markers such as aldehyde dehydrogenase (ALDH) enzymes are an intracellular target amenable of intervention that can also enable some level of treatment individualization. ALDH activity identifies CSCs in numerous cancers (Deng et al., 2010; Landen et al., 2010; Silva et al., 2011). Recently, Raghavan et al. demonstrated that ovarian cancer spheroids derived from cells that survived chemotherapy (cisplatin) displayed lower ALDH expression, complete loss of CD133 expression, and resistance to cisplatin/ALDH inhibitor combination treatment while spheroids that were resistant to cisplatin/JAK2 inhibitor combinations contained an increased number of ALDH^+^ cells (Raghavan et al., 2017; Chefetz et al., 2019). Thus, stratification of patients according to their CSC type (i.e., CSC markers and expression levels) pre- and post-therapy can potentially lead to more personalized treatment approaches (Patsalias and Kozovska, 2021). Ultimately this approach will not only allow us to better stratify patients for targeted and combinatorial therapies, but also potentially identify novel targets in CSCs.

## Conclusion

Targeting CSCs, as well as the phenotypic and functional heterogeneity of the bulk tumor cells derived from them, remains an unsolved clinical challenge. Phenotypic plasticity of CSCs fuels adaptive phenotypes that contribute to tumor chemoresistance and poor clinical outcomes. A second layer of complexity arises from spatially distinct CSC niches. In this review we focused on interrogating the tumor niche to zoom in on the resident CSCs and provide insights on potential pharmacological approaches. To date no scholarly review covers this specific topic under a unified systems biology framework and new insights on targeting CSCs and strategies to engineer TME are still limited. The use of ‘omics technologies as well as systems biology and computational methodologies has the potential to revolutionize the process of identifying and studying the CSC niche. While a wide array of methods have been developed to interrogate tumor heterogeneity, available methods still do not fully characterize the phenotypic states of CSCs or asses their plasticity and its impact on drug sensitivity. Therefore, there is an urgent need to develop tools that better integrate multimodal datasets to capture these cellular complexities. A crucial next step in the study of CSCs is to characterize their contributions to intratumoral heterogeneity, at various regulatory levels, from genotype to phenotype. If we are successful, the development of models to spatially localize CSC populations within tumors and dissect their contributions to tumor initiation, progression, and drug resistance will ultimately allow us to devise innovative strategies that target CSCs and improve cancer therapy.
